# Optimized PLGA encapsulated SA-2 nanosuspension exhibits sustained intraocular pressure reduction in the mouse microbead occlusion model of ocular hypertension

**DOI:** 10.1016/j.ejps.2025.107016

**Published:** 2025-01-17

**Authors:** Charles E. Amankwa, Biddut DebNath, Jennifer H. Pham, Gretchen A. Johnson, Wei Zhang, Amalendu Ranjan, Dorota L. Stankowska, Suchismita Acharya

**Affiliations:** a Department of Pharmacology and Neuroscience, University of North Texas Health Science Center, Fort Worth, TX 76107, USA; b North Texas Eye Research Institute, University of North Texas Health Science Center, Fort Worth, TX 76107, USA; c Department of Microbiology, Immunology and Genetics, University of North Texas Health Science Center, Fort Worth, TX 76107, USA

**Keywords:** PLGA nanoparticles, SA-2, intraocular pressure, primary open-angle glaucoma, bioavailability, retinal protection, sustained release

## Abstract

Elevated intraocular pressure (IOP) is implicated in the structural and functional damage to the retinal ganglion cells (RGCs) in primary open-angle glaucoma (POAG). Topical IOP lowering agents provide short-term relief, necessitating frequent dosing. Moreover, non-adherence to frequent eyedrops administration contributes significantly to visual field loss and worsens the disease outcome. We optimized the poly (lactic-co-glycolic acid) (PLGA) nanoparticles encapsulation of hybrid antioxidant-nitric oxide donor SA-2 (**SA-2NP**), investigated its bioavailability, duration of IOP lowering efficacy, and effects on retinal function in the microbead model of ocular hypertension (OHT).

SA-2 was bioavailable in the anterior and posterior segments after 1, 8, and 24 h post-single topical eyedrop administration. SA-2NP significantly lowered IOP (~25–34%) and preserved the RGC function after weekly eyedrop administration for 3 weeks in C57BL/6J mice. In conclusion, the optimized SA-2NP formulation demonstrated optimal bioavailability, ocular safety, and prolonged IOP-lowering efficacy in the mouse microbead occlusion model of OHT.

## 1. Introduction

Elevated intraocular pressure (IOP) is the primary risk factor for initiating and progressing primary open-angle glaucoma (POAG). It remains a cardinal parameter for POAG diagnosis and the only modifiable risk factor for managing POAG (Cohen & Pasquale, 2014). Although the pathogenesis of glaucoma is not fully understood, increased IOP is reported to exert mechanical stress and strain on the posterior structures of the eye, notably the lamina cribrosa and adjacent tissues. Intraocular pressure-induced stress causes compression, deformation, and remodeling of the lamina cribrosa, leading to degeneration of retinal ganglion cells (RGCs) and interruption of axonal transport (Cohen & Pasquale, 2014, Weinreb et al., 2014). Thus, IOP reduction is invariably firmly established as an effective treatment paradigm for slowing the progression of POAG.

To date, IOP-lowering medications, including cholinergic agents, carbonic anhydrase inhibitors (CAIs), β-adrenoceptor antagonists, and prostaglandin analogs (PGAs) essentially lower IOP by either increasing the outflow of aqueous humor (AH) or lowering AH formation (Addis & Miller-Ellis, 2018, Ondrejka et al., 2022). Similarly, surgical options, including trabeculectomy, glaucoma drainage implantation, and ciliary body cyclodestruction (for refractory glaucoma), all target IOP reduction (Aswin et al., 2024, Abdullatif & Ahmed El-Saied, 2021). While these procedures and therapeutics are effective at lowering IOP, long-term neurodegenerative damage and visual field loss persist (Bergea et al., 1999).

Previously, our group reported that the novel nitric oxide (NO) donating-antioxidant hybrid small molecule **SA-2** exhibited neuro-protective effects in three *ex vivo* and *in vivo* models of RGC injury, including *ex vivo* hypoxic stress in rat retinal explants, the IOP-independent optic nerve crush (ONC) model and the ischemia/reperfusion (I/R) model in mice. Treatment with **SA-2** conferred significant protection of RGCs in all models tested and further protected trabecular meshwork (TM) cells from oxidative stress-induced cell death (Stankowska et al., 2019, Amankwa et al., 2021, Amankwa et al., 2023, Amankwa et al., 2023). Topical ophthalmic therapy faces well-known challenges, including poor ocular bioavailability due to rapid precorneal elimination, systemic absorption, and limited corneal permeability. This often necessitates frequent dosing or high drug concentrations that may impact patient compliance. Emerging nanotechnology-based drug delivery systems, including Poly (D,L-lactic-co-glycolic acid) (PLGA) nanoparticles, represent promising candidates for ocular drug delivery (Bucolo et al., 2012, Xu et al., 2013). PLGA is approved by the FDA and widely utilized as a delivery platform for bioactive molecules. It has increased efficacy, reduced systemic side effects, and provided a more sustained drug release profile (Jiang et al., 2020, Khan et al., 2021, Swetledge et al., 2021, Varela-Fernandez et al., 2022).

Consequently, we developed the nano-encapsulated SA-2 (**SA-2NP**) formulation to improve the **SA-2** drug delivery and efficacy in ocular hypertensive rodents. A single topical ocular administration of **SA-2NP** resulted in a substantial reduction in IOP of approximately 45–50% within 3–6 h in the Ad5.TGFβ-2-induced mouse model of ocular hypertension (OHT) with the IOP lowering effect lasting for 30 h. Similarly, in Morrison’s rat model of OHT, a single topical eyedrop of **SA-2NP** lowered IOP significantly with a prolonged duration of action persisting for 72 h (Stankowska et al., 2021). While promising compared to the standard of care, some anti-glaucoma eyedrops are administered every 12 h or 24 h in the human eye for efficient IOP reduction. Our next goal was to improve the IOP-lowering effect of the single dose of **SA-2NP** eyedrop to longer than 72 h and possibly overcome the inherent limitations of topical ophthalmic drug delivery.

In the present study, we optimized the first-generation **SA-2NP** formulation to provide an optimal drug release profile and improve the duration of the IOP lowering efficacy in the microbead occlusion model of OHT following weekly administration of **SA-2NP** eyedrops. The optimized **SA-2**-loaded nanoparticles were characterized by assessing the mean particle size, zeta potential, polydispersity index, drug loading, and drug release assays. An ocular biodistribution study was conducted in mouse eyes following eyedrop dosing to assess the bioavailability of **SA-2** from the optimized **SA-2NP** formulation in both anterior and posterior ocular tissues. Additionally, we performed pattern electroretinography (PERG) and visual evoked potential (PVEP) to assess retinal function. We counted RBPMS-positive RGCs following treatment with **SA-2NP** in retinal flat mounts to determine RGC viability. Overall, this study demonstrates the efficacy of our in-house optimized **SA-2NP** formulation in improving the duration of action of the IOP lowering activity of the hybrid nitric oxide donor-antioxidant compound **SA-2** in the microbead occlusion model of OHT.

## 2. Materials and Methods

### 2.1. Materials and Reagents

Magnetic microsphere microbeads were purchased from ThermoFisher Scientific and Bangs Laboratory Inc (diameter-4.5 and 8.26 μm, Dynabeads^™^ M-450 Epoxy, ThermoFisher Scientific, Waltham, MA, USA) (Bangs Laboratory Inc, Fishers, IN, USA). Poly (lactic-co-glycolic acid) (PLGA, LG 50:50 acid endcap, Mn 25,000 -35,000 Da, PolySciTech, USA) and polyvinyl alcohol (Sigma Aldrich, St. Louis, MO, USA) were purchased and used for nanoparticle formulation synthesis. All the starting material and reagents required for the synthesis of compounds **SIN-1** and **SA-2** were purchased from Sigma Aldrich (St. Louis, MO, USA), and both compounds were synthesized and characterized in-house using our previously published protocol (Acharya et al., 2016).

### 2.2. Animals

Animal studies were performed in accordance with the Association for Research in Vision and Ophthalmology (ARVO) resolution for the Use of Animals in Ophthalmic and Vision Research and approved by the University of North Texas Health Science Center (UNTHSC) Institutional Animal Care and Use Committee (IACUC-2023–0005). C57BL/6J mice (male and female, 10–12 weeks) were used for all *in vivo* studies (*n* = 8–10 animals/group) and were purchased from the Jackson Laboratory (Bar Harbor, ME, USA). The mice were maintained on a constant dim light cycle with food and water available ad *libitum*.

### 2.3. Microbead Occlusion Model of Ocular Hypertension

For the microbead occlusion model, ocular hypertension was induced in one eye of C57BL/6J mice according to the protocol of Ito et al. (2016) with some modifications (Ito et al., 2016). However, in cases where the IOP elevation failed in the left eye, the right eye was used as an alternative. Mice were given an intraperitoneal injection of an anesthetic cocktail (10 mg/mL ketamine and 1 mg/mL xylazine) to induce deep general anesthesia, along with topical eyedrops of 0.5% proparacaine hydrochloride (Alcon Laboratories, Inc, Fort Worth, USA) to numb the eye. The pupils were dilated with 1% tropicamide (Somerset Therapeutics, LLC, Hollywood, FL, USA). 1.5 μL of 4.5 μm magnetic microbeads suspension in PBS (Dynabeads^™^ M-450 Epoxy, Thermo-Fisher Scientific, Waltham, MA, USA) was injected into the anterior chamber using a glass micropipette and a micro syringe pump (World Precision Instruments, Sarasota, FL, USA). A handheld magnet was used to reposition the microbeads towards the iridocorneal angle. Triple antibiotic ophthalmic ointment (Bausch+Lomb, Tampa, FL, USA) was applied after the procedure. Mice were kept warm on a heating pad until fully awake and were allowed to recover for 24 h before resumption of IOP measurements. The other eye was used as a contralateral control.

### 2.4. Fabrication and characterization of **SA-2 and SIN-1** polymeric nanoparticle (NP)

The fabrication and characterization of **SA-2NP** were conducted similarly to the previously described protocol with modifications (Hinkle et al., 2021). 10 mg of pure **SA-2** was dissolved in 100 μL dimethyl sulfoxide (DMSO) and transferred dropwise to 3.2 mL of chloroform containing 90 mg of PLGA to form an oil phase and stirred overnight. 20 mL of 2.5% polyvinyl alcohol (PVA) solution (water phase) was prepared and kept under constant moderate stirring for 5–7 minutes while adding the oil phase dropwise. Using a 10 mL scintillation vial, batches of 4 mL emulsion were sonicated at 70% for 1 min cycle with 30 seconds pulse per vial using Hielscher Ultrasound Technology UP 200S (Landsberger, Germany). The sonication cycle for each sample was repeated for 5 minutes per vial. The emulsion was stirred overnight under a vacuum to evaporate the organic solvent completely. Next, the nanosuspension was pelleted by ultracentrifugation at 17,000 rpm for 20 min (Beckman Coulter Inc. Life Sciences, USA), followed by washing three times with ultrapurified Milli-Q^®^-water (Millipore SAS, Molsheim, France). Finally, the nanoparticle was dissolved in 10% sucrose solution as a cryo/lyoprotectant and lyophilized to obtain a powder form for characterization (Koppolu et al., 2010, Menon et al., 2014, Shah et al., 2011). **SIN-1 NP** was prepared using a method similar to that of **SA-2 NP**.

A dynamic light scattering device determined the size (Supplementary Figure S1), polydispersity index, and zeta potential of SA-2NP (DLS, Malvern Zetasizer Ultra Particle Analyzer Co. Grovewood, UK). The supernatant collected from **SA-2NP** preparation was used to assess the amount of **SA-2** loaded in the nanoparticles using UV spectrometry (ʎ_max_ 300nm and 320nm for **SIN-1**). **SA-2NP** drug loading efficiency (Supplemental method S1) was calculated by the following Equation (1):

$$SA-2\;loading\;efficiency\;\left(\%\right)=\frac{\left(Initial\;SA-2\right)-\left(Unloaded\;SA-2\right)}{\left(Initial\;SA-2\right)}\times 100\%$$

The *in vitro* drug release profile was also determined using the molecular weight cut-off dialysis method (Supplemental method S2).

### 2.5. Eyedrops Administration and Intraocular Pressure Measurements

C57BL/6J mice (male and female, 10–12 weeks, *n* = 8–10 animals/group) were used in the study to evaluate the IOP-lowering efficacy of modified **SA-2NP** under a masked protocol. Prior to eyedrops administration, baseline IOP measurements were performed using a Tono Lab rebound tonometer (TonoLab; Icare; Helsinki, Finland) daily between 8:00–11:00 am as previously described (Stankowska et al., 2015), to establish baseline IOP without treatment. Three weeks after microbead occlusion surgery, baseline elevated IOP readings were measured, after which treatments were administered. In brief, topical eyedrops (5 μL) containing blank nanoparticles (**1% blank NP**), SIN-1 nanoparticles (**1% SIN-1NP** with 3% free drug)- positive control or **1% SA-2NP** with 3% free drug were administered in the IOP-elevated eye weekly for 3 weeks using the other eye as a contralateral control. IOP was measured and recorded after minimal sedation with 2.5% isoflurane via inhalation. IOP was calculated from an average of six consecutive IOP readings per sitting. The IOPs of the contralateral eyes were monitored until the end of the experiment, and IOP-lowering activity was expressed as an average difference in IOP between the treated and control eyes.

### 2.6. Pattern Electroretinography and Visual Evoked Potential (PVEP)

Pattern electroretinography (PERG) was used to measure the function of RGCs, and PVEP was used to assess the visual pathway by recording the amplitude and latency of the PERG/PVEP waveform following microbead occlusion surgery in mice, as described by Porciatti (Porciatti, 2015). Animals were anesthetized using an intraperitoneal injection of a cocktail of ketamine (10 mg/mL, Ketaved) and xylazine (1 mg/mL; Sigma Aldrich, St. Louis, MO, USA) to induce deep general anesthesia. The mouse was then placed on a heated stage. PERG responses were evoked in the dark environment in response to the contrast reversal of patterned visual stimuli using a commercially available PERG system (Jorvec Inc., Miami, FL, USA). The PERG responses were recorded from a needle electrode placed sub-dermally in the mouse snout, the reference electrode placed on the head’s base, and the ground electrode inserted at the tail’s base. Each animal was positioned at 11 cm from the display monitors. Stimuli (458 radii visual angle subtended on full-field pattern, two reversals per second, 300 averaged signals with cutoff filter frequencies of 1–30 Hz, 98% contrast, 800 cd/m2 average monitor illumination intensity) were delivered without dark adaptation to exclude the possible effect of direct photoreceptor-derived evoked responses. The PERG and PVEP amplitudes and latency at baseline and 21 days following post-treatment were measured and analyzed as previously described. (Minton et al., 2012)

### 2.7. Immunohistochemistry

To assess the potential toxicity or neuroprotective effect of **SA-2NP** in the retina, RGCs were quantified via flat mounts by immunostaining following the detailed protocol (Menon et al., 2014). The flat mounts were imaged using the Keyence BZ-X710 (Keyence Corporation, Osaka, Japan), and labeled RGCs were counted in a masked, manual manner using the cell counter plugin (http://imagej.nih.gov/ij/; version 1.54f provided in the public domain by the National Institutes of Health, (Bethesda, MD, USA). Twelve images were taken from peripheral, mid-peripheral, and central regions around four quadrants of each retina. The number of cells in four equal-sized fields (0.02 mm^2^ retina area) was counted and averaged (Minton et al., 2012). The data were presented as mean ± standard error of the mean (SEM), and the cell count was performed in a masked manner.

### 2.8. Nanosuspension Formulation and Storage

For eyedrop administration, **1% SA-2NP** with 3% free **SA-2** (weight/volume, *w/v*) was reconstituted in 1x PBS (pH_7.4_) and vortexed to obtain a milky white nanosuspension and the pH of the suspension was measured. The rationale for adding 3% free **SA-2** to the nanoformulation was to allow immediate IOP lowering action by free drug (<12 h time points) post-dosing of the nanosuspension eyedrop as the drug release from nanoparticle begins after 12 h at pH 7.4. 5 μL topical eyedrop of this formulated nanosuspension containing ~600 μg of **SA-2**/eyedrop was administered to mouse eyes for each dose, and the stock formulation was stored at −20°C till further use.

### 2.10. Ocular Biodistribution Study after **SA-2NP** Eyedrop Instillation

C57BL/6J mice (10–12 weeks, *n* = 2 animals/group) were used in the study to evaluate the biodistribution of SA-2 of the modified **SA-2NP** under a masked protocol. The tissues in the anterior segment (cornea+iris), lens, and posterior segment (choroid + retina + sclera tissues) were collected at 1 h, 8 h, and 24 h post topical eyedrop dosing (5 μL) of **SA-2** free drug plus modified nanosuspension 1% **SA-2NP** or 2% **SA-2NP** in mouse eyes. (Stankowska et al., 2019, Tse et al., 2017) The tissue samples were processed by adding 100 μL of cold PBS to prevent drying and homogenized on ice using a Fisher Scientific Tissue Homogenizer (Model: FB50) for 10–20 seconds per cycle. The homogenized tissue was centrifuged at 68,000 × g at 4°C for 20 minutes, after which 450 μL of acetonitrile was added to the pellet. The mixture was vortexed for ~2 minutes and centrifuged again under the same conditions. The supernatant was collected and stored at −80°C. The tissue extracts were concentrated using a SpeedVac (Vacufuge Plus), with initial 20-minute cycles repeated as needed. The concentrated samples were reconstituted using a 10:1 ratio of Milli-Q water to acetonitrile for LC-MS analysis. Calibration standards were prepared with 50% acetonitrile-water or 50% isopropyl alcohol water, supplemented with 0.1% formic acid. Before analysis, all samples and standards were filtered through 0.45-μm Acro disc filters (VWR, Radnor, PA, USA). Using a modified in-house developed Agilent Technologies 6460 Triple Quad (QQQ) LC/MS method, the concentration of compound **SA-2** was quantified (supplemental method S3).

### 2.11. Statistical analysis

The mean IOP data points per time interval were computed and analyzed using the Wilcoxon Signed-Rank Test at a 5% significance level to determine the difference between IOP reduction at baseline and treatment periods. Data were expressed as mean ± standard error of the mean (SEM) or standard deviation (SD). When three or more groups were used, statistical analysis was assessed using ANOVA followed by a post *hoc* test of pairwise multiple comparisons using either Dunnett or Holm–Sidak method on GraphPad Prism version 9.0 (San Diego, CA). A significant difference was considered where *P* values were ≤ 0.05.

## 3.0. Results

### 3.1. Physicochemical properties of **SA-2** and **SIN-1** nanoparticles

To optimize ocular drug delivery, physicochemical properties of the nanoparticles must be strategically developed to increase the bioavailability of drugs in the front and back of the eye to prolong drug efficacy. An ideal ophthalmic drug delivery should release the drug in a sustained manner and ensure prolonged corneal residence time. This study investigated physicochemical properties such as size, polydispersity index, drug loading, and drug release profile of optimized **SA-2NP** and **SIN-1NP** formulations. We reconstituted freshly lyophilized SA-2, SIN-1, or blank NP formulations and obtained a homogeneously dispersed milky nanosuspension. **SA-2NP** and **SIN-1NP** encapsulation efficiency was calculated as 87% and 84%, respectively (Table 1). Table 1 shows a uniform particle size distribution observed for all formulations, with an average particle size ranging from 192.0 nm to 284.7 nm (supplemental figure S1). Particle size below 300 nm is considered optimum for ophthalmic drug delivery, offering enhanced absorption and reduced irritation while remaining well within the eye’s tolerance for particles under 10 μm (Youshia et al., 2012). The zeta potential values for all formulations were in the range of −5 to −12 mV and represent a metric for determining the surface charge and potential stability of colloids and nanomaterial distribution in liquids. All formulations exhibited negative zeta potential values, indicating a net negative charge on their surfaces (Table 1). This net negative charge enhances electrostatic attractions, facilitating easier cation adsorption when the pH of the solution surpasses the isoelectric point of the nano adsorbents (Cegnar et al., 2004). The polydispersity index also reflects the homogeneity or heterogeneity of the nanoformulations. Based on the submicron particle size, a sample is termed size homogeneous if its PDI value ranges from 0.1–0.3. Thus, a PDI greater than 0.3 results in heterogeneity (Cegnar et al., 2004). Our findings show that the PDI for all the formulations varied from 0.07 to 0.21, as shown in Table 1, indicating a homogeneous, narrow size distribution that reveals higher nanoparticle stability.

**Fig. 1.**
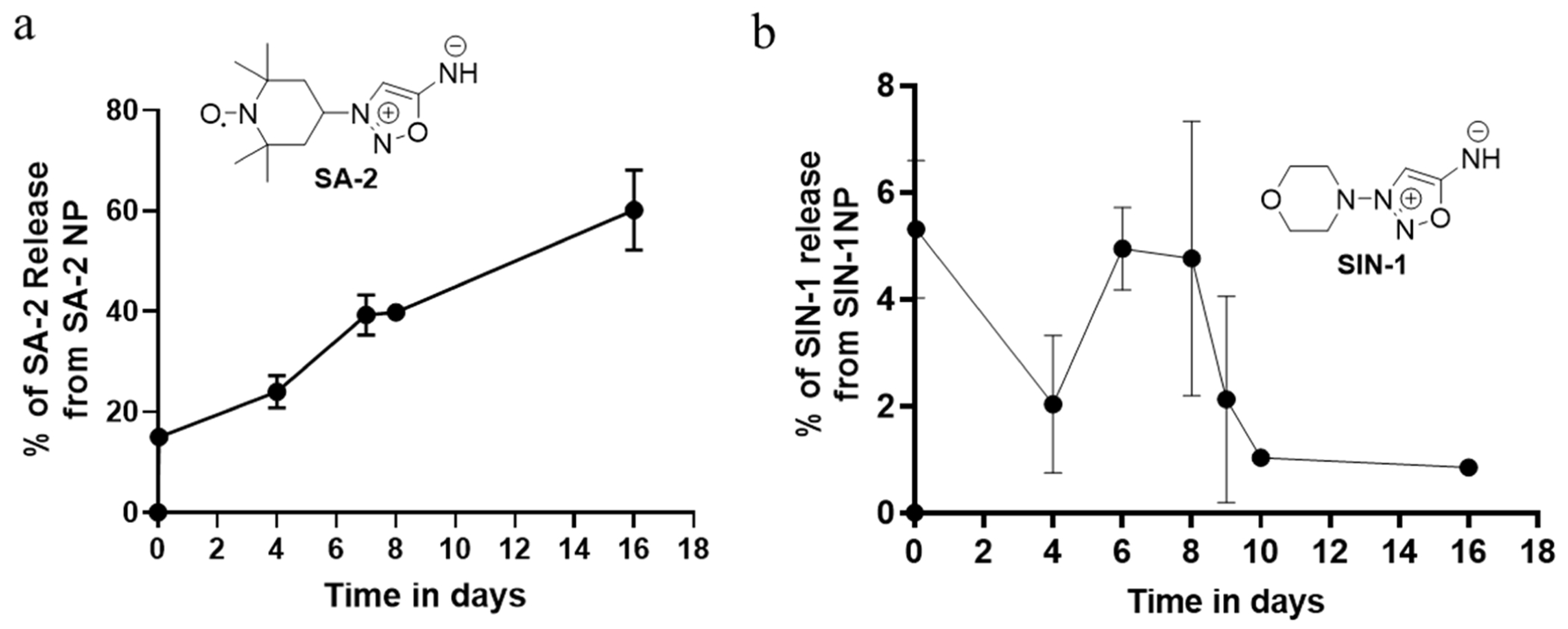
Percentage of drug release of SA-2 and SIN-1 from their corresponding nanoparticles in phosphate-buffered saline 7.4 (PBS_7.4_) (a) Drug release study of SA-2 from its PLGA-encapsulated nanosuspension **SA-2NP** in PBS at 37°C conducted for 16 days. The UV absorbance readings are taken in triplicates and quantified using the SA-2 standard curve at λ_max_ 300 nm. (b) A drug release study of SIN-1 from its PLGA-encapsulated nanosuspension **SIN-1NP** in PBS at 37°C was conducted for 16 days. The UV absorbance readings were taken in triplicates at λ_max_ 320nm. Data is presented as the mean ± SD of the triplicates.

*In vitro* drug release of **SA-2NP** and **SIN-1NP** was assessed via the indirect dialysis membrane molecular cut-off method (Fig. 1). **SA-2NP** showed an initial burst release of 14.5% within 1 h and a slow, sustained drug release of **SA-2** up to day 16. **SIN-1NP** demonstrated 5–6% burst release within 1 h and degraded faster at day 4, followed by another burst release at 6–8 days. From the drug release (%) *versus* time (in days) profile, **SA-2NP** showed higher drug release concentration than **SIN-1NP** (Figs. 1a,b). **SIN-1** is chemically unstable and degrades at 37°C within 15 minutes to an hour (Martin-Romero et al., 2004) and detected in lesser quantity than expected, whereas **SA-2** has a longer half-life of 2.3 days (supplementary figure S2). In addition, the relatively smaller particle size of the **SA-2NP** formulation decreases the surface area, subsequently lowering the diffusion path length. Moreover, the biodegradable polymer (PLGA) releases cargo in a controlled manner upon polymer erosion, thereby providing a protective barrier for **SA-2** against premature breakdown in the ocular microenvironment, further improving its stability (Khare et al., 2016).

### 3.2. **SA-2** is bioavailable in the posterior eye region following a single topical eyedrop administration of the **SA-2-NP** nanosuspension

**Fig. 2.**
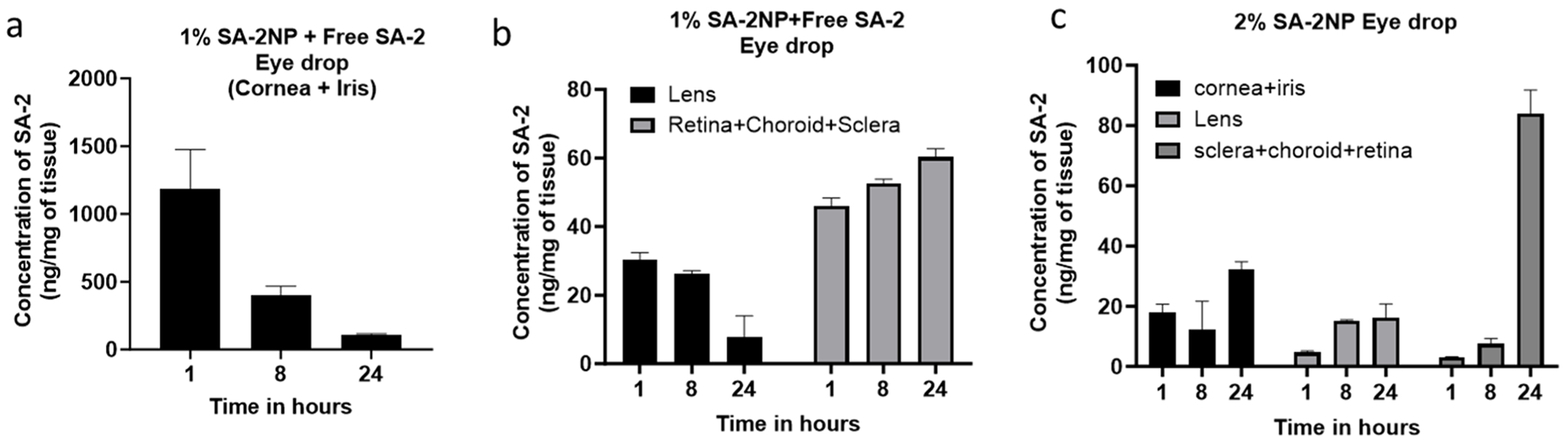
Biodistribution of **SA-2** in mouse ocular tissues after single topical administration of two different formulations (**1% SA-2NP** with free drug and **2% SA-2NP**) at 1 h, 8 h, and 24 h post-dosing. (a) The highest concentration of **SA-2** was detected in the anterior segment (cornea + iris) after 1 h and a time-dependent decrease to 401.5 ng/mg at 8 h and 106.2 ng/mg at 24 h (b) A slow and gradual decline in **SA-2** concentration was detected in the lens. However, the converse was observed in the posterior segment (retina + choroid + sclera). A time-dependent increase in the levels of SA-2 was detected in the **1% SA-2NP** + free drug formulation. c) The biodistribution profile of the nanosuspension without any added free drug (**2% SA-2NP)** showed distribution in the anterior segment, lens, and posterior region: n=4 tissues/time point. Data is presented as mean ± SD.

Previously, we reported that (Bucolo et al., 2012) **SA-2** was released from the first-generation **SA-2NP** formulation and was detected in quantifiable concentrations in cornea, retina, choroid, and sclera after a single topical eyedrop administration in mouse eyes (supplemental figure S3). Using the modified formulation, we conducted the biodistribution of **SA-2** following a single topical administration of two different formulations, namely the optimized **1% SA-2NP** with 3% of free **SA-2** (containing a total of 600 μg of **SA-2** combined from **1% SA-2NP** + 116 μg of free **SA-2**), and **2% SA-2NP (**437 μg of **SA-2)** formulations in ocular tissues. In this biodistribution study, we quantified the amount of compound **SA-2** in the anterior segment (cornea + iris), the lens, and in the posterior regions (retina, choroid, and sclera) following a single topical eyedrop application (5 μL) at 3 different time points (1 h, 8 h and 24 h). Compound **SA-2** was detected in sufficient concentrations in the posterior segment, the lens, and anterior segment tissue samples (45.97 ng/mg, 30.33 ng/mg, and 1184.75 ng/mg, respectively) as shown in Fig. 2, and data is summarized in Table 2.

### 3.3. Optimized **SA-2NP** eyedrop demonstrates prolonged IOP reduction in OHT mice eyes

**Fig. 3.**
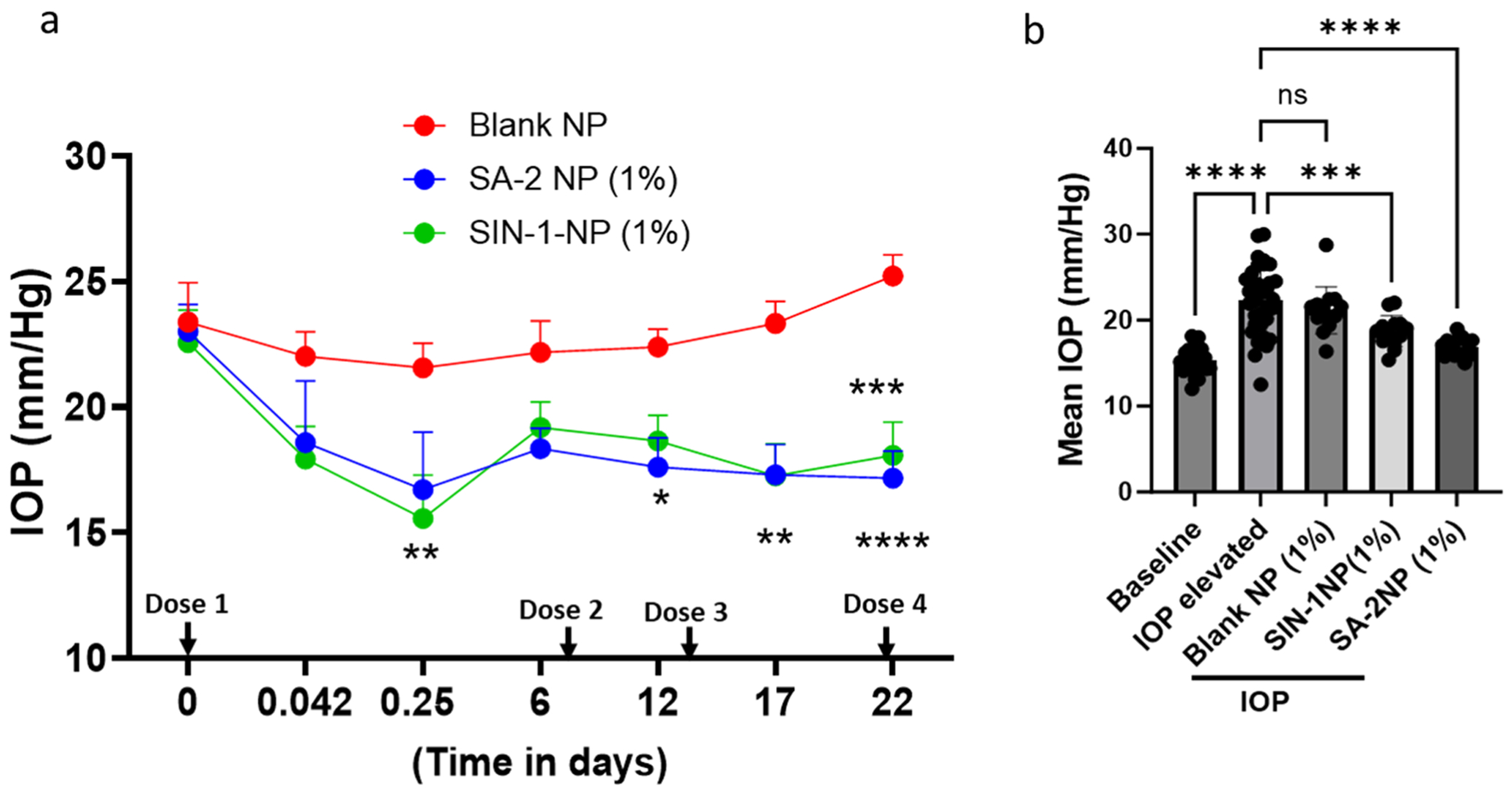
IOP-lowering effect of topically applied **SA-2** loaded NP (1% SA-2NP + 3% SA-2), **SIN-1 loaded NP** (1% SIN-1NP + 3% SIN-1), and **blank** NP in IOP elevated C57BL/6J mice eyes herein referred as IOP-elevated group. (**a**) The trace graph shows IOP data from Day 0-Day 22. Treatment with **SA-2NP** showed a sustained IOP-lowering activity in IOP-elevated mouse eyes with a significant decrease from days 12 and 17 through day 22 post-dosing (29.24%, ~6.9 mmHg, 21.40%, ~5.1 mmHg and 27.3%, ~6.45 mmHg decrease, respectively, compared to IOP elevated eyes, p≤.05, p≤0.01, p≤0.0001). **SIN-1NP-**treated mouse eyes demonstrated a burst IOP-lowering activity at 6 h with ~33% decrease in IOP when compared to IOP-elevated eyes (**b**) Data represents the change in mean IOP every 5–6 days from Day 1–22 after treatment. Compared to baseline IOP, microbead occlusion resulted in significantly elevated IOP readings, as shown by the IOP-elevated group (~7.8 mmHg, 53% increase, p<0.0001). Both **SA-2NP** and **SIN-1NP** significantly decreased IOP (25% to 31%, p<0.0001, 6.6 mmHg and 24% to 33%, p<0.001, 6.7 mmHg respectively) when compared to IOP-elevated eyes following topical administration from 1 h to day 22**.** Blank NP demonstrated no significant effect on IOP. All data were expressed as the mean ± SEM. GraphPad Prism, Two-way ANOVA for figure a; Tukey’s multiple comparison test, One way ANOVA for figure b. *p≤.05, ** p≤0.01, ***p≤0.001, ****p≤.0001 respectively (n = 10–14 eyes/group).

**Scheme 1.**
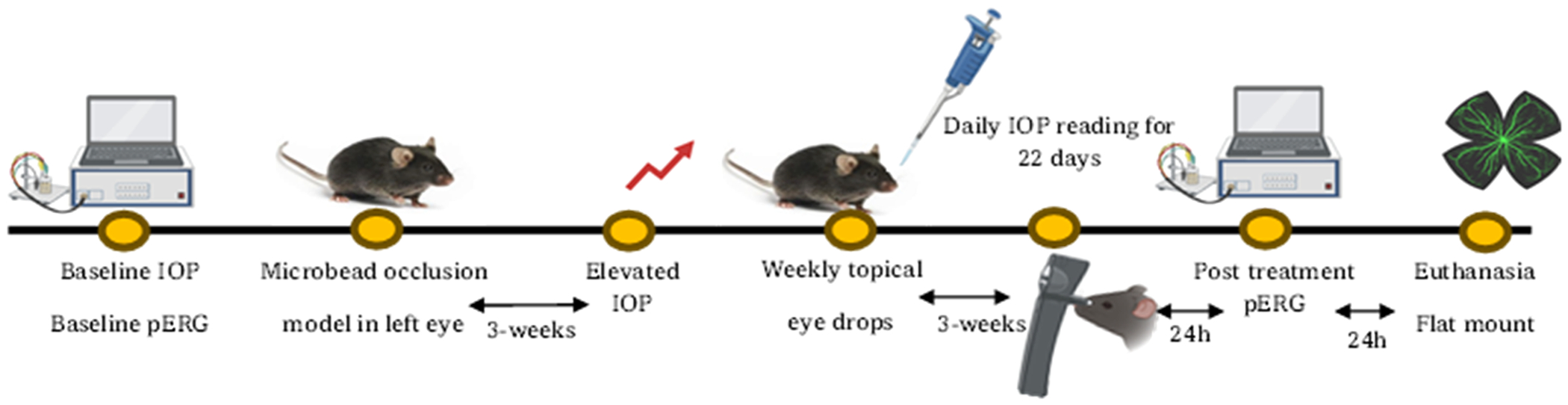
Schematic representation of *in vivo* study design for microbead occlusion-induced IOP elevation model in one eye of C57BL/6J mice.

Conventional topical ocular drug delivery systems must overcome many physical barriers, including corneal permeability, tear film barrier, reflex blinking, and nasolacrimal drainage. These barriers limit the passive absorption of diverse therapeutic molecules, thereby reducing their ocular bioavailability. To establish the efficacy of **SA-2NP** *in vivo*, we evaluated the IOP-reducing effect of **SA-2NP** and **SIN-1NP** in the microbead occlusion induced-IOP elevated C57BL/6J mouse eyes (Scheme 1). Blank NP, **SA-2NP** (with 3% SA-2, pH 6.36), or **SIN-1NP** (with 3% SA-2, pH 7.20) were instilled as single topical eyedrops on the IOP-elevated eyes weekly for three weeks. Compared to contralateral control, microbead occlusion (IOP-elevated group) resulted in a significant elevation of IOP, as shown in Fig. 3a. **SA-2NP**-treated mice had reduced IOP within the first hour after topical application of the first dose, and a sustained IOP-lowering profile was observed beginning as early as 6 hours post-dosing and throughout the three-week duration. Compared to blank NP-treated eyes, **SA-2NP** significantly decreased IOP at 6 h and on days 12, 17, and 22, respectively. As expected, due to the low metabolic stability of **SIN-1** (t½ = 0.5–1h) in physiological pH_7.4_ and its relatively faster nitric oxide release profile (Behar-Cohen et al., 1996), a steep and significant IOP reduction (~5 mm/Hg) was observed after 6 h post-dosing. However, the IOP increased gradually, peaking at day 6 before receiving the second dose. Additionally, weekly mean IOP readings measured post-second dose and from day 7 through day 22 showed that **SA-2NP** eyedrops had less variability in IOP change and were more efficacious (p≤0.001 *vs*. p≤0.0001) than **SIN-1NP** eyedrops as shown in Fig. 3b. As expected, there were no significant changes in mean IOP post-treatment with **blank NP** in the IOP-elevated group (Fig. 3).

### 3.4. Treatment with **SA-2NP** preserved RGC survival

**Fig. 4.**
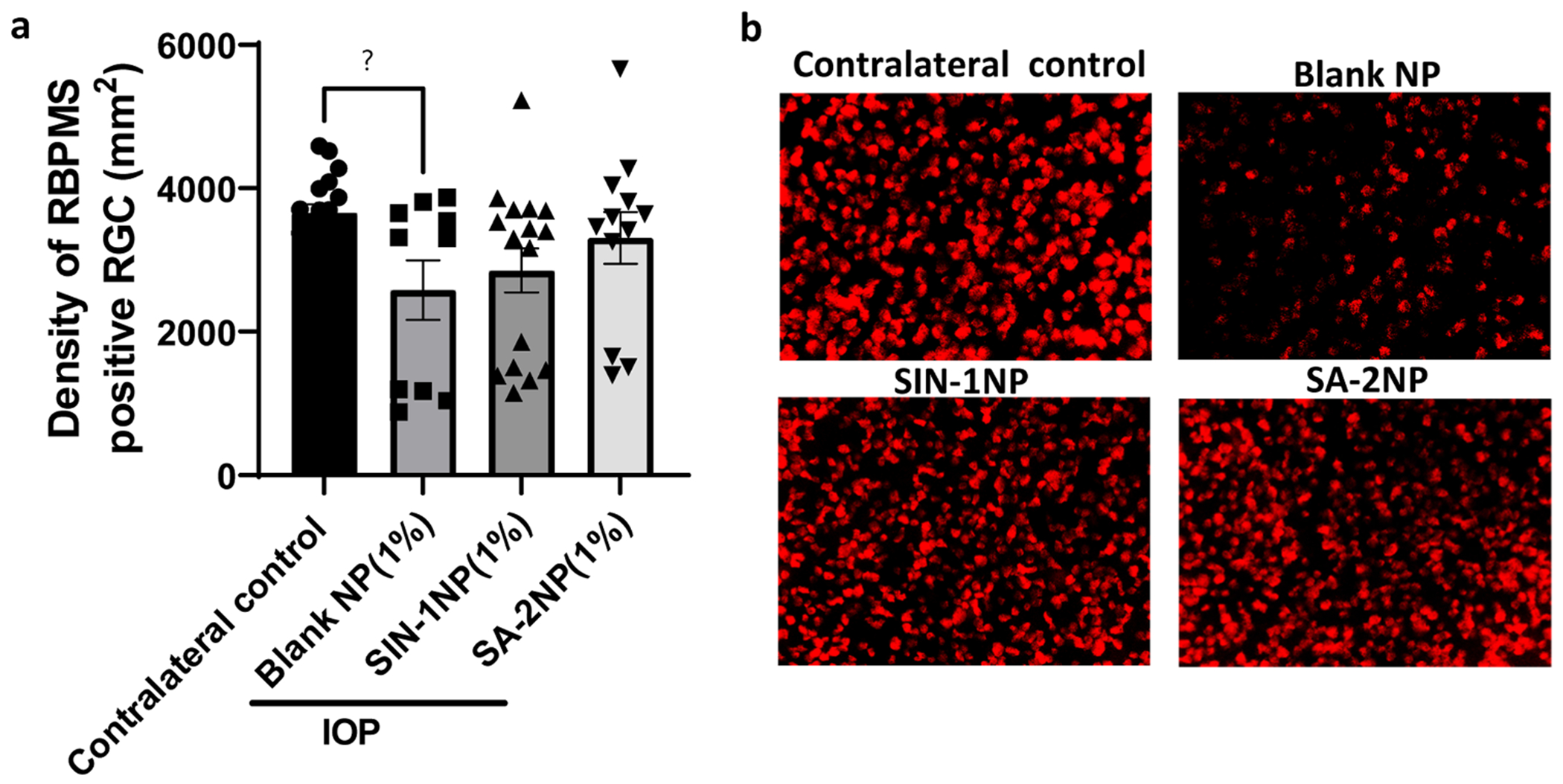
The **SA-2NP** and **SIN-1NP** formulations demonstrated the preservation of C57BL/6J retinal ganglion cells (RGCs). Retinal flat mounts from OHT-induced mouse eyes treated with either **SA-2NP** (1%) + 3% free SA-2, **SIN-1NP** (1%) + 3% free SIN-1, or **blank NP** (1%) were labeled with RBPMS, a marker for RGCs, in red, and were counted to determine RGC survival. (a) Quantification of total RGC counts per mm^2^ of retinal flat mounts. Microbead occlusion surgeries significantly decreased RBPMS-positive RGC counts (p≤0.05) in the blank NP group compared to the contralateral eyes. The **SA-2NP** and **SIN-1NP** groups showed no difference in RGC counts compared to contralateral counts, demonstrating preservation of RGCs after OHT insult. Additionally, the **SA-2NP-**treated group showed trends indicative of protection from microbead-induced damage to RGCs compared to the blank NP group. (b) Representative 40X images of RGCs treated with vehicle (**blank NP**), **SA-2NP**, or **SIN-1NP** in the presence of microbead occlusion-induced damage to RGCs. All groups were analyzed using One-way ANOVA, followed by Dunnett’s multiple comparisons test. Data represents the mean ± SEM. *p≤.05. GraphPad Prism. n = 7–10 eyes/group. The scale bar represents 200 μm.

Given that compound **SA-2** was bioavailable in the retina and other posterior tissues (Figs. 4a, b), we expected that **SA-2** would protect the retina from ocular hypertension-induced (OHT) damage and not exert any untoward neurotoxic effect on the retina. To demonstrate the neuroprotective effect of **SA-2NP** and **SIN-1NP**, we evaluated the number of surviving RBPMS-positive RGC counts following weekly administration of **1% SA-2NP**, **1% SIN-1NP**, or blank NP for 3 weeks in a masked manner. Unoperated eyes served as contralateral controls for the microbead occlusion model. RBPMS-positive retinal ganglion cell (RGC) counts were assessed at the retina’s central, mid-peripheral, and peripheral locations. As expected, blank NP-treated OHT-elevated eyes had a significant loss in RGC counts when compared to the contralateral control (29.57%, p≤.05) (Fig. 4a). Additionally, treatments with both **1% SA-2NP** and **1% SIN-1NP** showed trends indicative of improved RGC survival when compared to blank NP-treated eyes (28.39% and 10.73% increased preservation, respectively) (Fig. 4a). This suggests that both **1% SIN-1NP** and **1% SA-2NP** formulations are not neurotoxic to the RGCs and could be safe therapeutic candidates for managing retinal neurodegeneration.

### 3.5. Extended use of **SA-2NP** eyedrops showed no evidence of functional RGC deficits or retinal neural activity determined by PERG and PVEP

**Fig. 5.**
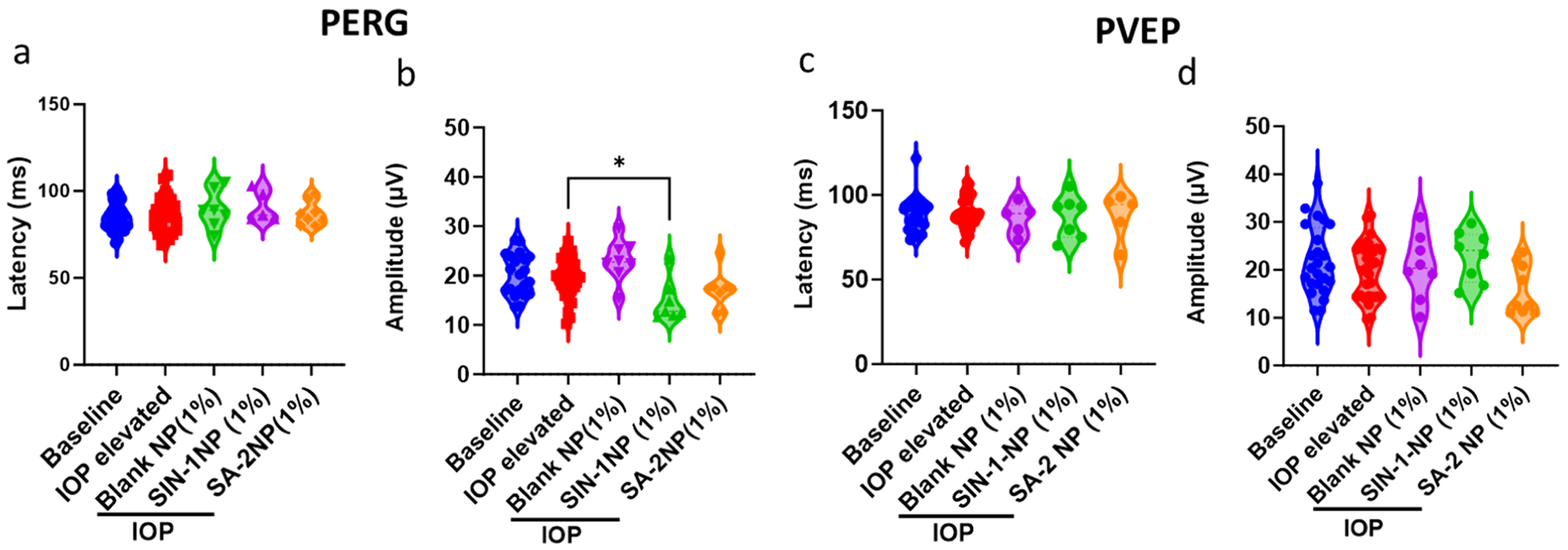
Weekly topical eyedrops administration (3 weeks) of **SA-2NP** (1% w/v) to C57BL/6J mouse eyes showed no functional RGC deficits. (a) Analysis of PERG latency in mice exposed to microbead occlusion and treated with either **SIN-1NP, SA-2NP** or blank NP. (b) The graph represents analyzed PERG amplitudes of mice treated with **SIN-1NP, SA-2NP,** or blank NP. PERG amplitudes of **SIN-1NP**-treated mouse eyes showed a significant decrease compared to those of IOP-elevated mice, suggesting an attenuated response to a visual stimulus or an overall decline in retinal function (*p < 0.05). GraphPad Prism, One-way ANOVA, Dunnett’s multiple comparisons test (n = 10–14 eyes/group).

We evaluated the effect of weekly topical instillation of **1% SA-2NP** on RGC function following the microbead occlusion-induced OHT in mouse eyes. Baseline PERG was determined prior to the microbead injection, before the dosing (IOP-elevated eyes), and post-treatment PERG was analyzed 24 h after the last day of IOP reading to determine the tolerability and therapeutic efficacy of **SA-2NP** eyedrops in preventing the decline in PERG amplitude. Interestingly, the IOP-elevated eyes treated with blank NP had no significant decrease in PERG amplitude. In contrast, a significant reduction in amplitude was observed after treatment with **1% SIN-1NP** (*p≤.05) compared to the amplitude of the IOP-elevated eyes with no treatment (Fig. 5b). This could potentially suggest a dysfunction or damage to the RGC post-treatment with NO donor **SIN-1** released from the nanoparticles. For **SA-2NP**, both the amplitude and latency of PERG remained unchanged compared to baseline PERG, indicating that compound **SA-2NP** did not adversely affect the electrical responses generated by the retina in response to visual stimuli (Figs. 5a, b) when compared to the electrical stimulus from IOP-elevated eyes. Pattern Visual Evoked Potential (PVEP) showed no changes in amplitude and latency in both **SIN-1NP-** and **SA-2NP**-treated mice. This suggests that **SA-2NP** and **SIN-1NP** may not alter the neural processing along the visual pathway from the retina to the visual cortex (Figs. 5c, d).

## 4. Discussion

The intricate anatomical and physiological barriers, including tear film, cornea, conjunctiva, reflex blinking, and nasolacrimal drainage, contribute to the limited bioavailability of topical medications: typically, less than 5% (Akhter et al., 2022). To overcome these ocular barriers, improve patient compliance, and limit frequent dosing, nanotechnologies provide the platform to achieve sustained/controlled drug release in ocular tissues (Onugwu et al., 2023, Agogo-Mawuli Percy & Siderovski, 2022). This study aimed to optimize and characterize **SA-2-**encapsulated nanoparticles to achieve sustained therapeutically effective drug delivery to the anterior and posterior eye segments in an experimental mouse model of ocular hypertension.

The current standard of care (SoC) for topical glaucoma therapies decreases IOP by reducing aqueous humor (AH) production or increasing AH drainage via the uveoscleral pathway (Rao & Epstein, 2007, Singh & Singh, 2023). Although reduction of IOP is essential, IOP-lowering alone does not fully mitigate the inherent susceptibility of RGCs and the optic nerve regions from neurodegeneration and death. Thus, a comprehensive therapeutic approach should encompass IOP modulation and direct neuroprotective strategies to address the multifaceted pathophysiology of glaucoma. Furthermore, an additional benefit will be to develop a drug delivery platform that ensures a prolonged and sustained release of medication, leading to a more consistent IOP reduction and enhanced patient adherence to the treatment regimen. Patient non-adherence remains a challenge in topical ocular drug delivery. Approximately 10% of visual field defects are attributed to non-adherence. Interestingly, forgetfulness in daily medication administration was cited as a primary contributing factor, thereby exacerbating the progression of the disease and increasing the risk of adverse outcomes (Konstas et al., 2000, Welge-Lussen et al., 2015).

Polymeric nanoparticles (NP) have emerged as one of the most viable drug delivery systems that improve tissue-specific drug targeting and increase bioavailability across biological membranes. In particular, poly (lactic-co-glycolic acid) (PLGA) has been approved by the Food and Drug Administration (FDA) for human use. It is extensively utilized for achieving modified-controlled release and site-specific drug targeting. Previously, we showed that the hybrid nitric oxide (NO) donor-antioxidant compound **SA-2** formulated into a slow-release nanoparticle suspension (**SA-2NP**) increased aqueous outflow facility, decreased episcleral venous pressure, and antioxidant effects (Stankowska et al., 2021). In addition, a single eyedrop of the first-generation **SA-2NP** formulation lowered IOP for up to 3 days in the mouse Ad5.TGFβ-2 and rat Morrison’s models of OHT, with quantifiable **SA-2** concentrations in ocular tissues (Stankowska et al., 2021). In the current study, **SA-2** delivered from the optimized NP formulation was detected in the cornea, retina + choroid, and the lens following a single topical administration. This suggests that **SA-2** from the new formulation was released sufficiently and available in ocular tissues. Several studies have corroborated the findings of the ability of PLGA NP to penetrate through the tear film and passively diffuse through the cornea and scleral pathway, potentially extending to the vitreous humor and retina (Gonzalez-Pizarro et al., 2018, Silva-Abreu et al., 2018, Silva-Abreu et al., 2018) with an average size ranging between 190–300 nm. Moreover, PLGA-based NP formulations have demonstrated the ability to protect active drugs from deactivation by cytochrome P450 enzymes found in the tear film, corneal epithelium, and ciliary body (Guengerich, 2017). This protective effect prolongs the drug’s half-life, improves therapeutic efficacy, and improves patient compliance.

The ratios of lactide to glycolide (LA/GA ratios) and the molecular weights (MWs) of PLGA can influence the NP characteristics, including particle size, biodegradation rate, and corneal permeation capabilities. Consequently, we developed PLGA NP with LA/GA ratios set at 50:50 and molecular weights ranging from 25kDa to 35kDa and analyzed the physicochemical properties. The size range for the **SA-2NP** (200–300nm) in PBS fell within the acceptable criterion for topical ocular drug delivery systems, indicating a lower risk for irritation (Ali & Lehmussaari, 2006). Moreover, the polydispersity index (PDI) values for all formulations were characteristic of a monodispersed system with a low probability of aggregation (PDI ≤ 0.21). The negative zeta potential values observed are attributed to the characteristics of the PLGA polymer, primarily due to the terminal carboxylic acid groups present in the polymer chain. (Patel & Agrawal, 2011)

As expected, the *in vitro* drug release pattern of **SA-2** in PBS at 37°C was highly correlated with the *in vivo* IOP-lowering efficacy. *In vitro*, the drug release profile for 1% **SA-2NP** demonstrated a nearly 15% **SA-2** release after 1 h with a prolonged steady increase up to ~64% over day 16. Concurrently, **SA-2NP** formulation significantly lowered IOP in the microbead occlusion model of OHT mouse eyes, with prominent effects observed at 6 h, days 3, 6, 16, and 22 post-dosing. SIN-1 (linsidomine) is a known NO donor with a short half-life (<1 h in biological pH at 37°C), and its carbamate prodrug form: molsidomine is an approved drug for the management of angina pectoris via oral dosing. When formulated as an eyedrop, **SIN-1** lowers IOP in OHT rabbit eyes; however, the effect lasts up to only 3 h (Behar-Cohen et al., 1996). In contrast to the drug release *in vitro*, we observed that the IOP lowering efficacy was similar for both **SA-2NP** and **SIN-1NP** in OHT mouse eyes. This could be attributed to the high affinity of the drug to its polymeric matrix, which may contribute to the slow diffusion of the drug, allowing for a sustained and prolonged release (Allahyari & Mohit, 2016). Compared to IOP-elevated control eyes, **SA-2NP** significantly decreased IOP in 6 h and by ~31% (7.5 mm/Hg) during treatment. The IOP-lowering effect was sustained until day 22, corroborating the results we showed for the bioavailability in ocular tissues and drug release profile in PBS formulation. The significant IOP-reducing effect and acceptable safety profile of **SA-2NP** make it a promising candidate for further studies in larger preclinical animal models to increase clinical translatability and potentially delay the onset of neurodegeneration. However, we also observed marginal reduction in IOP in the blank NP group. This unexpected finding suggests that aliphatic polyester polymer, like PLGA, may possess inherent IOP-lowering properties. Lactic and glycolic acids’ degradation products could interact with receptors in the trabecular meshwork or uveoscleral pathway, enhancing aqueous humor outflow. Further research is needed to elucidate this mechanism and its implications. The translatability of these results is influenced by differences in ocular anatomy (*e.g*., corneal thickness), species-specific variations in drug metabolism and clearance, and the complexities of dose scaling from mice to humans, including adjustments for surface area, ocular volume, and systemic exposure. Empirical evidence indicates that in POAG, increased IOP precipitates distinct compositional and structural changes in the ONH, which may directly/indirectly trigger the neurodegeneration and death of RGCs via apoptosis (Pitha et al., 2023). Therefore, the survival and protection of RGCs from apoptosis are crucial to maintaining the function and morphology of the retina. Since the RGCs play a pivotal role in transmitting visual information from the eye to the brain, an ideal topical ocular therapeutic agent should effectively deliver drugs to the target sites without causing cytotoxic or neurotoxic effects on RGCs. Treatment with **SA-2NP** eyedrops to OHT mouse eyes improved RPBMS-positive RGC counts by 36.37% relative to the group treated with 1% blank NP. While **SIN-1NP** showed a similar trend in protecting RGCs, a significant reduction in pattern electroretinogram (PERG) amplitude was observed, suggesting the RGCs in **SIN-1NP**-treated eyes had a loss of neural functionality.

## 5. Conclusion

In summary, the optimized **SA-2NP** formulation possessed high encapsulation efficiency, improved drug release profile with no self-aggregation, and an average size appropriate for ocular administration (< 300nm). The relevance of this work is highlighted by the sustained IOP lowering effect of **SA-2NP** lasting nearly 6 days after a single eye-drop administration with no overt visual toxicity and protecting RGCs from death. These findings underscore the potential of utilizing optimized **SA-2NP** as a promising pharmacological candidate for topical anti-glaucoma therapy. By achieving prolonged IOP reduction, this formulation may enhance patient compliance and advance the current non-invasive treatment strategies for POAG. Further studies involving pharmacokinetic and dose-response studies in large animal eyes are essential to ensure the clinical translatability of **SA-2**.

## Supplementary Material

1

Supplementary material associated with this article can be found, in the online version, at doi:10.1016/j.ejps.2025.107016.

## Figures and Tables

**Table 1:** Characterization of the nanoparticles.

Sample	Particle size (nm± SD)	PDI	Zeta potential (mV ± SD)	Encapsulation efficiency (%)
SA-2NP	232.3 ±4.7	0.097	−4.98 ± 0.72	87.39
SIN-1NP	284.7 ± 3.40	0.21	−4.69 ± 1.08	84.16
Blank NP	192.2 ± 1.4	0.073	−11.66 ± 0.13	N/A

**Table 2:** Bioavailability of **SA-2** in the mouse ocular tissues.

1% SA-2NP + free drug	Cornea + Iris (ng/mg)	Lens (ng/mg)	Retina + choroid + Sclera (ng/mg)
**1 h**	1184.7 ± 289.8	30.3 ± 2.1	46.0 ± 2.4
**8 h**	401.5 ± 65.7	26.4 ± 1.0	52.5 ± 1.3
**24 h**	106.2 ± 10.7	7.8 ± 6.1	60.3 ± 2.4
2% SA-2NP	Cornea + Iris (ng/mg)	Lens (ng/mg)	Retina + choroid + Sclera (ng/mg)

**1 h**	17.9 ± 2.9	4.7 ± 0.4	3.0 ± 0.3
**8 h**	12.2 ± 9.5	15.3 ± 0.2	7.5 ± 1.8
**24 h**	32.3 ± 2.4	16.2 ± 4.5	83.9 ± 7.9

## Data Availability

The raw/processed data required to reproduce these findings will be available upon request.
