# Supplementary material for: Optimized PLGA encapsulated SA-2 nanosuspension exhibits sustained intraocular pressure reduction in the mouse microbead occlusion model of ocular hypertension
Source: Eur J Pharm Sci. Author manuscript; Available in PMC 2026 May 16. (PMC13179673; doi:10.1016/j.ejps.2025.107016)
Supplement: 1 [file NIHMS2170155-supplement-1.docx]

**SUPPLEMENTARY DATA**

**Supplemental Methods**

**S.1. Drug loading**

A fixed amount of nanoparticle solution (2 mg/mL) was added to a transparent cuvette and placed in the instrument. The nanoparticle properties were detected by laser light scattering produced by Brownian motion of the particles. High-resolution size distributions and number-based concentrations were assessed using NanoSight Nanoparticle Tracking Analysis (NTA) version 3.4 Build 3.4.4 (Malvern Panalytical Limited, UK). **SA-2NP** drug loading efficiency and drug release profile were quantified by measuring UV absorbance for **SA-2** (ʎmax = 300 nm) via Cytation5 Microplate reader. For **SA-2NP** drug loading efficiency, free **SA-2** was diluted in a phosphate-buffered saline (PBS) at different concentrations to generate an **SA-2** standard curve.

**S.2. *In vitro* drug release**

To adequately estimate **SA-2NP** drug release profile, 1 mL of **1%** **SA-2NP** with 3% of free **SA-2** suspension (5 mg/mL) in 1x PBS pH was placed in a dialysis bag with molecular weight cut off (MWCO 3.5–5 kDa (Spectrum, Catalog 131192), submerged in 20 mL 1x PBS p (dialysate) and shaken at a speed of 100 rpm at 37°C for 30 days. At each time point (1 h, 3 h, 6 h, 12 h, 24 h, 48 h, 3 days, to 30 days), an aliquot of 1 mL of dialysate solution was withdrawn and replaced with the same volume of fresh PBS to maintain sink conditions. The withdrawn aliquot (1 mL) was filtered through a 0.45 μm membrane. Each sampling solution was then read for the drug concentration via UV-visible spectroscopy using a BioTek Cytation 5 microplate reader (BioTek Instruments Inc., Highland Park, Winooski, VT, USA), and the amount of released **SA-2** was quantified using UV absorbance and calibrated against the **SA-2** standard curve (ʎmax = 300 nm). Consequently, a cumulative release profile of **SA-2** over time was plotted. **SIN-1NP** were characterized using a protocol similar to that of **SA-2NP**.

**S3. Bioanalytical method development to quantify SA-2 by High-Pressure Liquid Chromatography/Mass Spectroscopy (HPLC/LC/MS)**

Agilent Triple Quad (QQQ) LC/MS instrument (Agilent 6460 series, Agilent Technologies, Germany) was used to quantify the drug concentrations in ocular samples throughout the study. The triple-quadrupole mass spectrometer (TSQ Quantum ULTRA, Thermo, USA) was interfaced via an ESI probe with a liquid chromatograph (LC-20AD, Shimadzu, Japan). Chromatographic separation was achieved on an Atlantis HILIC Silica column (50 mm × 2 mm, 3 µm). A binary gradient of 0.1% formic acid in acetonitrile and ammonium formate was used, with the latter being 2% in 0–2 min, 50–50% in 2–5.5 min, and 2% in 5.6–7.0 min at a flow rate of 0.3 µL/min, and the column temperature was maintained at room temperature. The mass spectrometer was operated in the positive electron spray ionization mode, and quantification was performed using multiple reaction monitoring of the transitions from *m*/*z* 241.16→ *m*/*z* 214.16 for compound **SA-2**. The collision energy was set as 10 eV and the spray voltage 500 V. Both the vaporizer and the capillary temperatures were kept at 380°C. All samples were mixed with solvents of the mobile phases, filtered, and then 5 μL was injected into HPLC-MS for analysis. The peak **SA-2** concentration was calculated following the preparations of **SA-2** standard curves using the Agilent 6460 Technologies Mass Hunter Acquisition Software. The standard curves were generated from nine different concentrations of **SA-2** (0-250 ng/mL, correlation coefficient >0.99745). The detection limit was below 0.5 ng/mL and was within the linear limits of detection. Similarly, the limit of quantitation was 2.5 ng/mL.

**S4. Stability of SA-2 in PBS at different pH**

Mass of newly synthesized SA-2 was found 241.1660 (m/z), formula C_11_H_20_N_4_O_2_ in LC-TOF-MS with agilent C18 column in positive mode, and 241.20 (m/z) in LC-QQQ-MS tested with either reverse phase column or normal phase column. Here, the accelerated stability study of SA-2 at pH 6.4 and 7.4 was conducted at 40^o^C at different time points, and the samples were analyzed using LC-QQQ-MS with the same instrument method for the previous study (S3). Each sample was prepared and injected three times following standard curves. 100% purity for standards is assumed to be used.

**Supplemental Figures**

**
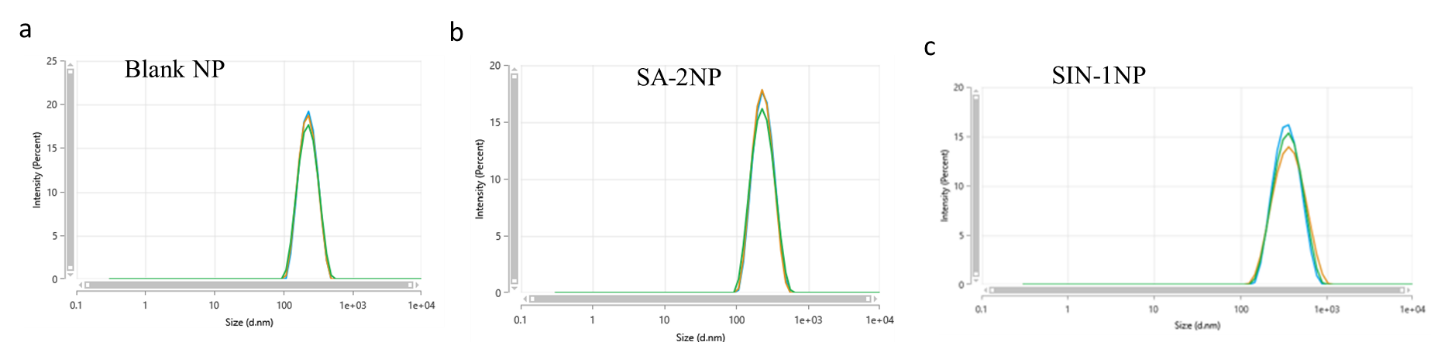
**

**Supplementary Figure S1:** Dynamic Light Scattering (DLS) curves showing particle size distribution for (a) Blank NP, (b) SA-2NP, and (c) SIN-1NP.

**Supplemental Figure S2:** Stability of **SA-2** in PBS at pH 6.4 and 7.4 conducted at 40^o^C at different time points for 28 days (4 weeks). From the data, we can see a SA-2 degradation trend. It is much more stable under acid conditions at pH 6.4. The half-life (t_1/2_) at pH 7.4 was calculated to be 2.3 days at pH 7.4 and 14 days at pH 6.4.

**
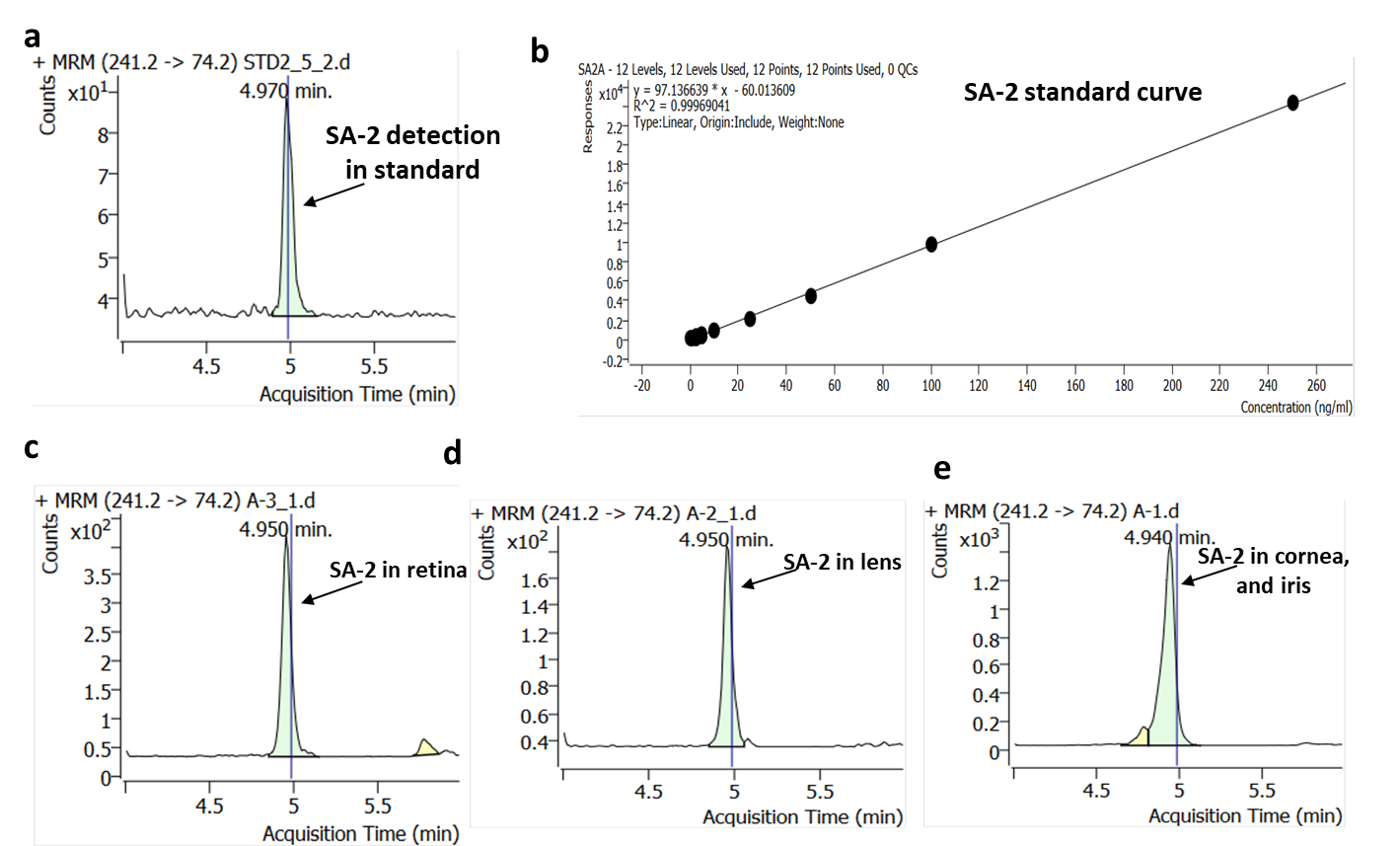
Supplementary Figure S3:** Quantitative detection of **SA-2** biodistribution in ocular tissues (a) Chromatogram of SA-2 detected in SA-2 standard (b) Standard curve of **SA-2** following chemical preparation in a previously established HPLC/MS diluent (c-e) Chromatogram of SA-2 detection in ocular tissue samples, retina, lens, and cornea+iris respectively.
